# Rice-Flour-Based Diet Is Associated with Reduced Obesity-Related and Glucose-Related Outcomes and Increased *Ucp3* Expression in KK-*A^y^* Mice

**DOI:** 10.3390/foods15183209

**Published:** 2026-09-10

**Authors:** Yoko Yokoyama, Nana Osari, Tanon Taworntawat, Sumito Sekimoto, Kazuo Tsubota, Naho Kitamura, Mitsuhiro Watanabe

**Affiliations:** 1 Graduate School of Media and Governance, Keio University, 5322 Endo, Fujisawa 252-0882, Kanagawa, Japan; 2 Health Science Laboratory, Keio Research Institute at SFC, Keio University, 5322 Endo, Fujisawa 252-0882, Kanagawa, Japan; 3 Tsubota Laboratory, Inc., 35 Shinanomachi, CRIK E7, Shinjuku-ku 160-8582, Tokyo, Japan; 4 Faculty of Environment and Information Studies, Keio University, 5322 Endo, Fujisawa 252-0882, Kanagawa, Japan; 5 Department of Internal Medicine, Keio University School of Medicine, 35 Shinanomachi, Shinjuku-ku 160-8582, Tokyo, Japan

**Keywords:** rice-flour-based diet, wheat-flour-based diet, body weight, glucose tolerance, *Ucp3* mRNA expression, skeletal muscle

## Abstract

This study investigates whether, in a mouse model under ad libitum feeding conditions, substituting a wheat-flour-based diet with a rice-flour-based diet influences body-weight- and glucose-related outcomes. Using female KK-*A^y^* mice, we compared diets containing wheat or rice flour. After seven weeks, the rice-flour-based diet was associated with significantly lower body weight (8.5% lower) and improved glucose tolerance compared with the wheat-flour-based diet; however, no significant improvement in insulin sensitivity was observed. Gene expression analysis revealed that *Ucp3* mRNA expression in skeletal muscle was significantly higher in the rice-flour-based diet group than in the wheat-flour-based diet group, whereas no significant differences were observed in the expression of genes encoding enzymes involved in pyruvate oxidation and the TCA cycle. These results suggest that the lower body weight and improved glucose tolerance observed in the rice-flour-based diet group may be associated with increased skeletal muscle *Ucp3* mRNA expression; however, whether this increase reflects altered UCP3 protein activity or energy expenditure remains unclear and requires further functional validation. Overall, substituting the wheat-flour-based diet with the rice-flour-based diet was associated with lower body weight and improved glucose tolerance in this model.

## 1. Introduction

The global prevalence of obesity is estimated to reach 14% among men and 18.5% among women, having more than doubled worldwide between 1990 and 2022 [1]. Furthermore, obesity is associated with severe complications such as diabetes, cardiovascular disease, and various cancers, directly impacting healthy life expectancy and quality of life [2,3]. Therefore, addressing and preventing obesity are recognized as a major global health challenge.

Previous studies indicate that sustaining 10% weight loss is effective in improving complications [4], making it a key therapeutic target. Recent reviews of obesity treatment suggest that lifestyle modifications and behavioural interventions can achieve this 7–10% weight loss target [5,6,7], but they also highlight the difficulty of maintaining such weight loss over the long term [4].

In obesity and dietary therapy research, meta-analyses comparing diets with varying nutritional balances have shown that multiple dietary approaches—such as low-carbohydrate or low-fat diets—all produce weight loss effects [8]. However, the differences in weight loss between individual diets are minimal, indicating that no single macronutrient ratio is dramatically superior [8]. The key factor is whether a dietary approach can be maintained over the long term [4]. As a staple food and a major component of diets, grains inevitably account for a high proportion of total energy intake [9]. Therefore, an approach that involves changing the staple grain-based dietary pattern rather than restricting intake may offer advantages in terms of sustainability. Indeed, wheat and rice account for more than half (50–70 percent) of global dietary energy intake [10], and clarifying differences in metabolic outcomes between wheat-based and rice-based dietary patterns may have relevance to sustainable dietary strategies.

In recent years, a large-scale ecological study covering 170 countries worldwide reported that countries with a high supply of wheat have higher rates of obesity (a positive correlation), while countries with a high supply of rice have lower rates of obesity (a negative correlation) [11]. Furthermore, a large-scale cohort study in China has also revealed that while wheat intake is associated with obesity, among men, a higher intake of rice is linked to a reduced risk of obesity [12]. The results of these observational studies in humans suggest that higher rice intake may be associated with more favourable obesity-related outcomes than higher wheat intake.

However, although these observational studies adjust for potential confounding factors such as gender, age, socioeconomic background, physical activity, and total energy intake, they have limitations in that they cannot completely rule out the influence of unmeasured confounding factors, and it is difficult to analyse the direct effects on the various organs involved in metabolism, along with the mechanisms of these effects. In contrast, the use of mouse models makes it possible to examine, in detail, the effects on metabolism-related organs under conditions where genetic and environmental factors are strictly controlled.

Previous studies have suggested that differences in the structure of starch in rice and wheat, as well as variations in the types and content of dietary fibre and resistant starch, may be associated with their obesity-suppressing effects. In the field of diabetes and related areas, consumption of low-glycaemic index (GI) foods is considered effective for maintaining blood glucose control [13,14]; however, the GI values of rice and wheat vary depending on factors such as the state of processing [15] and physical form (e.g., powder or grains) [16,17,18], and consistent results have not been obtained.

Therefore, the aim of this study was to use both rice and wheat in powdered form—thereby standardizing the influence of physical form—and to investigate differences in body-weight- and glucose-related outcomes between rice-flour-based and wheat-flour-based diets. The KK-*A^y^* mouse model was selected as it exhibits phenotypes similar to those seen in human obesity and type 2 diabetes [19]. Through this study, we aimed to investigate whether substituting a wheat-flour-based diet with a rice-flour-based diet was associated with differences in body weight and glucose tolerance in KK-*A^y^* mice and to examine its association with insulin sensitivity.

## 2. Materials and Methods

### 2.1. Materials and Reagents

Polished rice grains (*Oryza sativa* L.; cultivar: Koshihikari; batch no.: 221021-8) were kindly provided by Pentofork Co. Ltd. (Fukui, Japan). The rice grains were harvested in Ishibashi-cho, Fukui, Japan, and underwent primary selection using a 1.9 mm mesh sieve. They were then subjected to secondary selection using an optical colour sorter and polished. Commercially refined wheat flour (*Triticum aestivum* L.) was purchased from a local supplier (Nisshin Seifun Group Inc., Tokyo, Japan). Before preparation of the diet, both the rice grains and the wheat flour were heated and subsequently freeze-dried. The rice was cooked in a domestic rice cooker with a capacity equivalent to five Japanese rice cooker cups; typically, approximately five cups of rice were used per batch. The wheat flour was mixed with water, transferred to a microwave-safe food container, and heated in the microwave for approximately 5 to 10 min. If it was judged, by visual inspection, that the heating was insufficient, additional heating was carried out. After heating, both ingredients were freeze-dried. This heating procedure was not a controlled heat treatment intended to modify the properties of the starch; rather, it was carried out to prepare ingredients in a cooked form that broadly represents human consumption patterns. The precise temperature profile, the ratio of sample to water, the total heating time and the cooling conditions were not specified as controlled experimental variables. The freeze-dried samples were then finely ground using a laboratory mill (Force Mill, Osaka Chemical Co., Ltd., Osaka, Japan). Particle size was estimated by taking microscopic photographs of the ground powder placed on a microscope slide using an Olympus CKX41 inverted microscope with a ×10 objective lens (Evident Corporation, Tokyo, Japan). Calibration was performed using the 200 µm grid on a WATSON disposable cell-counting plate (FUKAEKASEI Co., Ltd. and WATSON Co., Ltd., Tokyo, Japan), after which the equivalent circular diameter was calculated. The mean (median) equivalent circular diameter was 50.92 (50.41) µm for rice flour and 44.19 (42.14) µm for wheat flour, calculated on the basis of 319 and 220 particles, respectively. As only one image from a single sample of each type of flour was analysed, these measurements were used solely for descriptive characterization. The diet for mice was prepared using this flour. The composition of the diet is shown in Table A1. The same purified sources were used for protein and fat in each group, and these were standardized across the high-fat-diet groups. The same amounts of dietary fibre, vitamins, and minerals were added to each group. The resistant starch content values of rice flour and wheat flour were 0.6 g/100 g and 1.7 g/100 g, respectively. Other nutritional data are shown in Table A2.

### 2.2. Animals and Experimental Design

Five-week-old KK-*A^y^* female mice (obese and diabetic model mice [19,20]) were purchased from CLEA Japan Inc., Shizuoka, Japan. Mice were kept at room temperature between 23 °C and 25 °C on a 12 h light/dark cycle with food and water available ad libitum. After 1 week of environmental acclimatization, random allocation was used to generate five experimental groups, each composed of six mice. The five groups were a low-fat and higher-carbohydrate (corn-starch- and sucrose-based diet) group (LFHC-CS; 10% kcal fat, 80% kcal carbohydrate), a high-fat and higher-carbohydrate (corn-starch- and sucrose- based diet) group (HFHC-CS, 45% kcal fat, 45% kcal carbohydrate), a high-fat and higher-carbohydrate (wheat-flour-based diet) group (HFHC-W, 45% kcal fat, 45% kcal carbohydrate), a high-fat and lower-carbohydrate plus relatively higher protein (wheat-flour-based diet) group (HFLC-W, 45% kcal fat, 35% kcal carbohydrate), and a high-fat and higher-carbohydrate (rice-flour-based diet (Koshihikari)) group (HFHC-R, 45% kcal fat, 45% kcal carbohydrate). To compare the rice-flour-based diet and wheat-flour-based diet, a high-fat diet (45% kcal fat) containing wheat flour (HFHC-W) was used as a control group for the rice-flour-based high-fat diet (HFHC-R). Next, to compare the high-fat diet (45% kcal fat) with the standard diet (10% kcal fat), we included a high-fat diet (HFHC-CS) and a standard diet (LFHC-CS) using corn starch and sucrose—ingredients commonly used in standard mouse feed—as dietary composition control groups. Furthermore, to compare wheat-flour-based diets with different carbohydrate and protein proportions, an HFLC-W group was included specifically within the wheat flour comparison group in which the carbohydrate content was reduced by 10% kcal and the protein content was increased by 10% kcal.

In terms of husbandry conditions, 6 individuals were housed in each cage, and each was identified by ear-punching. We followed a protocol prepared before the study to clean the cages, measure the animals’ body weights and food intake, and monitor mice on a weekly basis.

**Figure 1:**
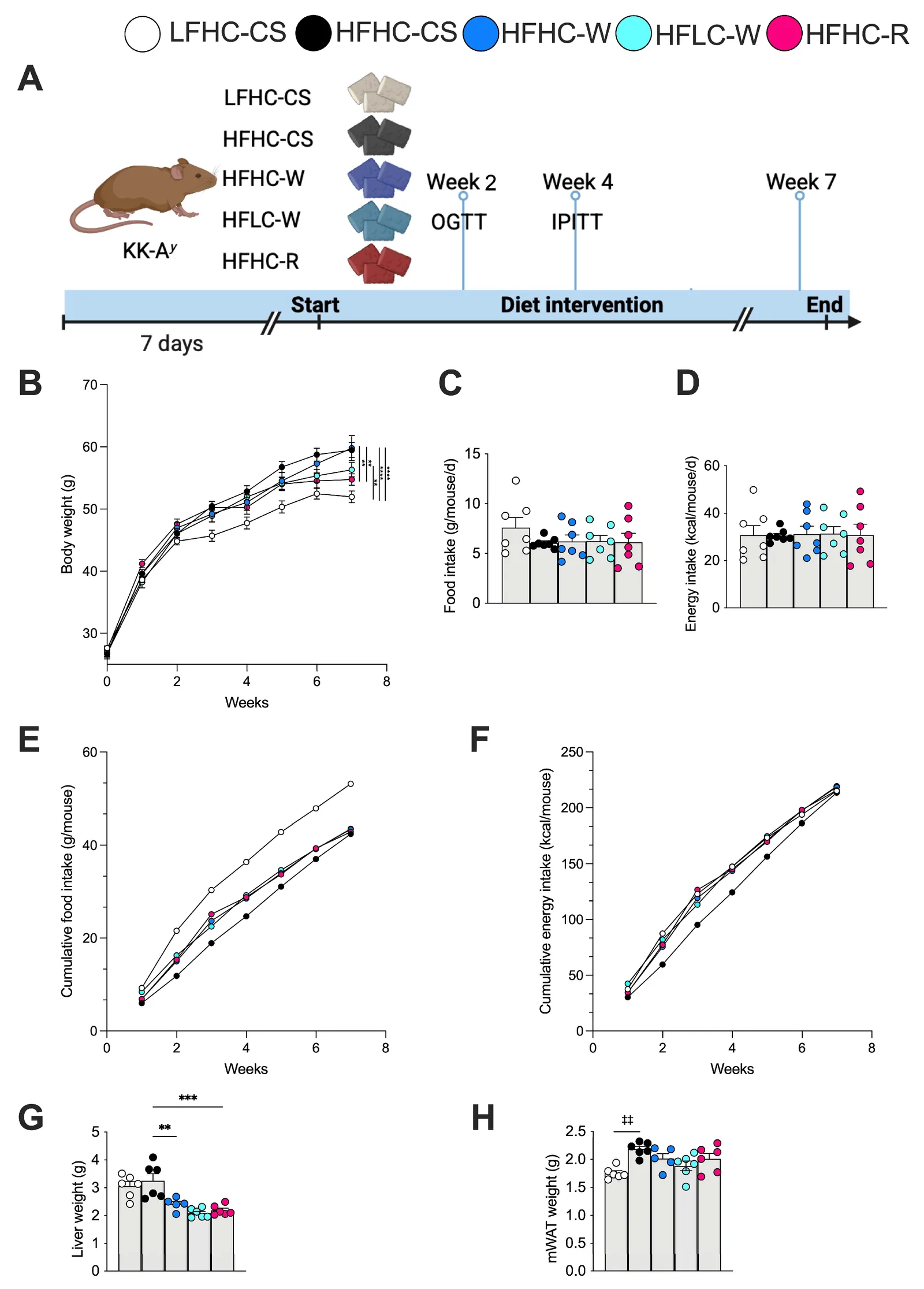
Body weight, food intake, liver weight, and mesenteric white adipose (mWAT) tissue weight in KK-*A^y^* mice fed the experimental diets. (**A**) Study design (created with BioRender.com). (**B**) Change in body weight across the different groups (*n* = 6 mice/group up to week 6; thereafter, *n* = 5 for the HFHC-W group and *n* = 6 for all other groups). (**C**) Estimated mean daily food intake per mouse under ad libitum feeding conditions. (**D**) Estimated mean daily energy intake per mouse. (**E**) Estimated cumulative food intake per mouse. (**F**) Estimated cumulative energy intake per mouse. Food intake was measured at the cage level (one cage per group), and per-mouse values were estimated based on the number of mice housed during each period. No statistical comparisons were performed for panels (**C**–**F**). (**G**) Liver tissue weight (*n* = 5 for the HFHC-W group, and *n* = 6 for all other groups). (**H**) mWAT weight (*n* = 5 for the HFHC-W group, and *n* = 6 for all other groups). LFHC-CS, low-fat higher-carbohydrate diet using corn starch and sucrose; HFHC-CS, high-fat higher-carbohydrate diet using corn starch and sucrose; HFHC-W, high-fat higher-carbohydrate diet using wheat; HFLC-W, high-fat, lower-carbohydrate, relatively higher-protein diet using wheat; HFHC-R, high-fat higher-carbohydrate diet using rice. Except for the descriptive food- and energy-intake estimates in Figure 1C–F, data are presented as the mean ± s.e.m. ** *p* < 0.01, ‡‡ *p* < 0.01, *** *p* < 0.001, and **** *p* < 0.0001. *p* values were calculated using analysis of variance, assessed by Tukey’s multiple comparisons test. The comparison symbols indicate the following comparisons: * HFHC-R versus HFHC-W or HFHC-CS; and ‡ LFHC-CS versus HFHC-CS.

The study design is shown in Figure 1A. The exclusion criteria were established a priori in the study protocol. During routine monitoring in week 6, one mouse in the HFHC-W group was found to have an injury that met one of the prespecified exclusion criteria and was therefore withdrawn from the study. Data collected before the injury was detected were retained because they had been obtained before the exclusion event and met the criteria for inclusion in the analysis; no data were collected from this animal thereafter. Consequently, the HFHC-W group comprised *n* = 6 up to week 6 and *n* = 5 thereafter and for terminal analyses. The exact sample size for each endpoint is provided in the corresponding figure legends.

To negate confounding factors, the measurements and dissections were carried out on one animal from each group in ascending order, starting with the first group and working through to the last. This procedure was then repeated with one animal from each group in descending order.

Seven weeks after the start of treatment, animals were fasted for 6 h and dissected. The animals were euthanized by inhalation of isoflurane. In addition to blood, visceral organs, including the liver, mesenteric white adipose tissue (mWAT), and skeletal muscle (gastrocnemius), were removed, and the weights of the liver and mWAT were measured. Liver and mWAT tissues were collected from the same sites in each individual and used for subsequent histological processing and microscopic evaluation. The collected blood and organs were rapidly frozen in liquid nitrogen and stored in a freezer at −80 °C.

### 2.3. Body Weight, Food Intake, Liver and Adipose Tissue Measurements

Body weight and food intake were measured weekly. Weekly food consumption was calculated by subtracting the weight of food remaining in the food rack at the end of each one-week period from the weight of food provided at the beginning of that period. Any food spilled within the cage was collected and weighed separately, and this amount was subtracted from the apparent food consumption. Food intake was measured at the cage level, with one cage per dietary group. Estimated daily intake per mouse was calculated by dividing weekly cage consumption by the number of mice housed during the corresponding period and by seven days. The weights of the liver and mWAT were recorded at the time of dissection.

### 2.4. Glucose Tolerance and Insulin Sensitivity Measurements

Two weeks after the start of treatment, an oral glucose tolerance test (OGTT) was performed using mice fasted for 4 h. Glucose was administered orally at 1.5 g/kg; blood was collected by tail vein at 0, 30, 60, 90, and 120 min; and blood glucose was measured using Niprostat Strip XP3 (NIPRO Corporation, Osaka, Japan).

Four weeks after the start of treatment, an intraperitoneal insulin tolerance test (IPITT) was performed on mice fasted for 6 h. Insulin (Humulin N, Eli Lilly and Company, Indianapolis, IN, USA) was administered intraperitoneally at 1.25 U/kg, and blood glucose levels at 0, 30, 60, 90, 120, and 180 min were measured using the Niprostat Strip XP3 (NIPRO Corporation, Osaka, Japan).

### 2.5. Haematoxylin and Eosin (H&E) Staining

Liver tissue (the central part of the largest lobe) and mWAT collected at autopsy were fixed with Tissue-Tek Ufix (Sakura Finetek Japan Co., Ltd., Tokyo, Japan) and embedded in paraffin. After sectioning, tissues were stained with H&E. After deparaffinization and rehydration, the sections were stained with haematoxylin and eosin, dehydrated, cleared, and mounted according to the standard protocol. The stained sections were digitized using a NanoZoomer-XR slide scanner (Hamamatsu Photonics, Shizuoka, Japan) and evaluated histologically. Investigators were blinded during histological evaluation.

### 2.6. Plasma and Liver Metabolic Parameter Assay

Lipid extraction was performed using the Folch method [21]. After drying, the lipid extracts were re-dissolved in 2-propanol containing 10% Triton X, and each parameter was measured.

Plasma and liver triglyceride (TG) concentrations were determined enzymatically using a commercially available assay kit (Determiner L TG II; Kyowa Medex, Tokyo, Japan). Plasma samples and liver extracts were incubated with the kit reagents at 37 °C according to the manufacturer’s protocol. After completion of the enzymatic colour reaction, absorbance was measured at 600 nm using a microplate reader (Multiskan FC; Thermo Fisher Scientific Inc., Waltham, MA, USA). TG concentrations were calculated from the absorbance of the TG standard and normalized to the plasma volume or liver tissue weight, as appropriate.

### 2.7. Gene Expression Analysis

Total RNA was extracted from frozen samples using the acid guanidinium thiocyanate–phenol–chloroform extraction (AGPC) method [22]. Total RNA of skeletal muscle was extracted using the RNeasy Fibrosis Mini Kit (Qiagen, Hilden, Germany). cDNA was synthesized from total RNA using PrimeScript RT Master Mix (Takara Bio, Inc., Shiga, Japan). Quantitative RT-PCR measurements of individual cDNAs were performed on a QuantStudio 5 (Thermo Fisher Scientific Inc., Waltham, MA, USA) using THUNDERBIRD SYBR qPCR Mix (TOYOBO, Co., Ltd., Osaka, Japan). The mRNA expression levels in liver and skeletal muscle were corrected for gene expression levels of the housekeeping genes *β-actin* and *18S*. The sequences of the primers used in this study are listed in Table A3.

### 2.8. Resistant Starch

Resistant starch concentrations were determined enzymatically using a commercially available assay kit (Resistant Starch Assay Kit, K-RSTAR; Megazyme International Ireland Ltd., Wicklow, Ireland). Samples were incubated with pancreatic α-amylase and amyloglucosidase at 37 °C according to the manufacturer’s protocol, and the resistant-starch fraction was recovered and enzymatically hydrolysed to glucose. After completion of the glucose oxidase–peroxidase colour reaction, absorbance was measured at 510 nm using a microplate reader (Multiskan FC; Thermo Fisher Scientific Inc., Waltham, MA, USA). Resistant starch concentrations were calculated from the absorbance of the D-glucose standard and normalized to the dry weight of the sample.

### 2.9. Statistical Analysis

Except for the descriptive food- and energy-intake estimates in Figure 1C–F, data are presented as the mean ± s.e.m. Statistical analysis was performed using GraphPad Prism 10 (GraphPad Software, Boston, MA, USA). For the oral glucose tolerance test (OGTT) and intraperitoneal insulin tolerance test (IPITT), the area under the curve (AUC) was calculated using the trapezoidal rule as the primary summary measure. Differences in AUC among the groups were evaluated using one-way analysis of variance (ANOVA), followed by Tukey’s multiple-comparisons test. Time-course blood glucose concentrations during the OGTT and IPITT were analysed using two-way repeated-measures ANOVA, with treatment, time, and their interaction included as factors, followed by Tukey’s multiple-comparisons test at individual time points. Thus, correlations among repeated measurements obtained from the same animal were accounted for in the analysis.

For other single-point parameters, including liver and plasma metabolic parameters and gene-expression levels, one-way ANOVA followed by Tukey’s multiple-comparisons test was used. No adjustment for multiple testing across the different genes was applied, because the RT-PCR analyses were exploratory and hypothesis-generating.

Potential outliers in the gene-expression datasets were assessed using the robust regression and outlier removal (ROUT) method in GraphPad Prism, with the false-discovery rate (Q) set to 1%. One value in the HFLC-W group of the *Mcp1* mRNA expression dataset was identified as an outlier and excluded before statistical analysis. No other datapoints were excluded. No observations were missing from any of the datasets, including the OGTT and IPITT time-course data; therefore, no missing-data imputation was performed.

The significance level was set to *p* < 0.05. In the graphs, the values are indicated as follows: * *p* < 0.05; ** *p* < 0.01; *** *p* < 0.001; and **** *p* < 0.0001. The comparison symbols indicate the following comparisons: * HFHC-R versus HFHC-W or HFHC-CS; † HFHC-W versus HFLC-W; and ‡ LFHC-CS versus HFHC-CS.

## 3. Results

### 3.1. Body Weight, Food Intake, Liver Weight, and Mesenteric White Adipose Tissue Weight in KK-A^y^ Mice Fed the Experimental Diets

Body weight was significantly higher in the HFHC-CS group (59.48 g ± 1.20) than in the LFHC-CS group (51.95 g ± 0.93) (*p* < 0.0001) (Figure 1B). Body weight was 8.5% lower in the HFHC-R (54.73 g ± 0.92) group than in the HFHC-W group (59.80 g ± 2.04) (*p* = 0.0061) (Figure 1B). No statistically significant difference in body weight was observed between the HFLC-W and HFHC-W groups (Figure 1B).

The estimated mean daily food and energy intakes per mouse were broadly similar among the groups (Figure 1C, D). When comparing cumulative food intake, we found that the HFHC-W, HFHC-R and HFLC-W groups showed broadly similar trends, lying between the LFHC-CS and HFHC-CS groups (Figure 1E). When comparing cumulative calorie intake, we noted that the HFHC-CS group tended to have lower values, whilst no clear differences were observed among the other groups within the range of the data shown (Figure 1F). Liver weight was significantly lower in the wheat-flour- (*p* = 0.0081) and rice-flour (*p* = 0.0004)-based diet groups compared with the starch/sucrose-based diet group (Figure 1G). The mWAT weight increased in the high-fat diet group (HFHC-CS) compared to the low-fat diet group (LFHC-CS) (*p* = 0.0019) but did not differ in the other comparisons (Figure 1H). 

### 3.2. Glucose Tolerance and Insulin Sensitivity in KK-A^y^ Mice Fed the Experimental Diets

**Figure 2:**
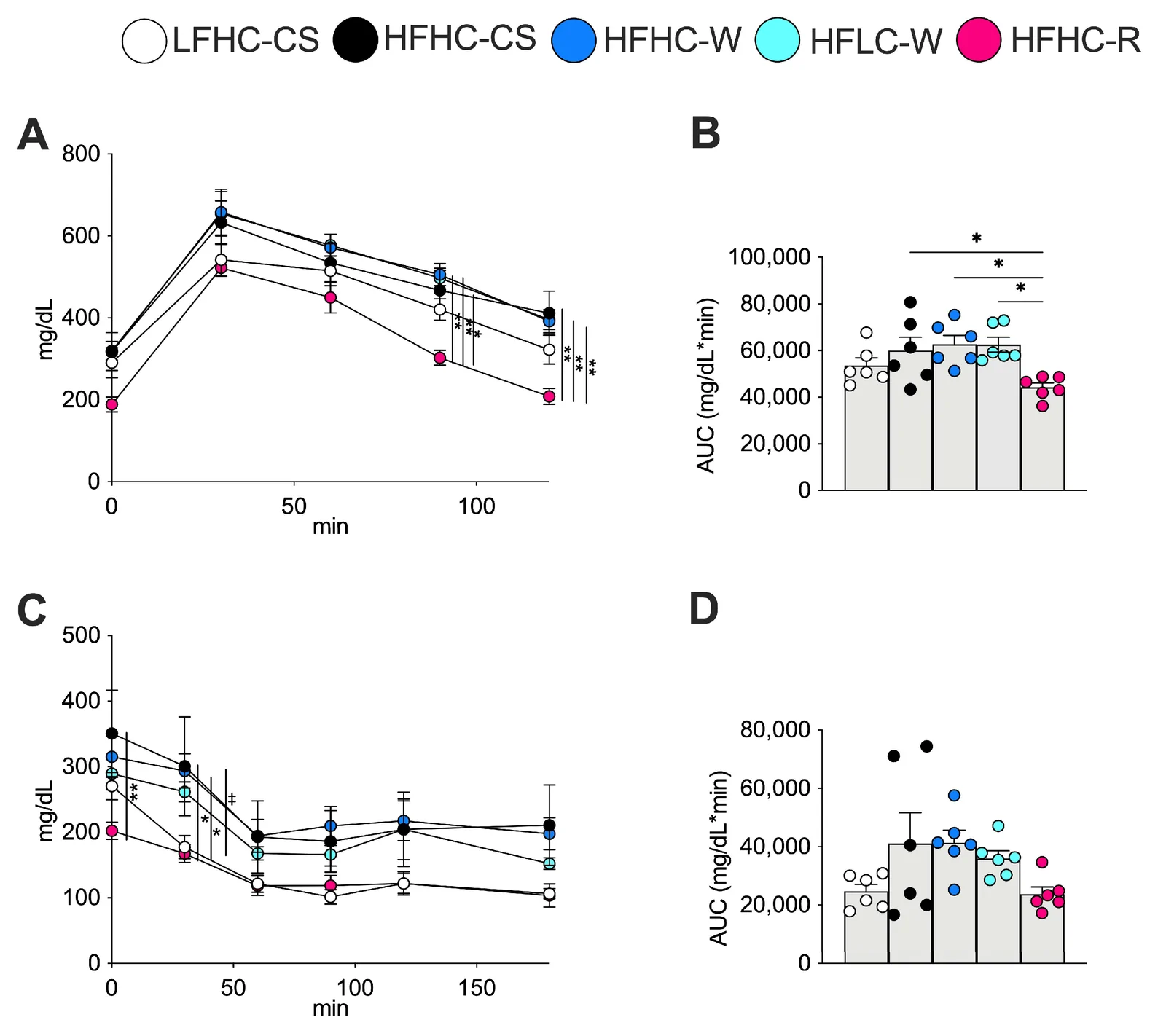
Glucose tolerance and insulin sensitivity in KK-*A^y^* mice fed the experimental diets. (**A**) Oral glucose tolerance test (OGTT), 2 weeks after initiation of dietary treatment (*n* = 6 mice/group). Glucose was administered by gavage at a dose of 1.5 g/kg of body weight after 4 h fast. (**B**) Area under the curve (AUC) of the glucose excursion during the OGTT in panel A (*n* = 6 mice/group). (**C**) Intraperitoneal insulin tolerance test (IPITT) after 4 weeks of treatment after 6 h fast (*n* = 6 mice/group). Insulin was injected at a dose of 1.25 U/kg of body weight. (**D**) AUC of the glucose levels during IPITT (*n* = 6 mice/group). LFHC-CS, low-fat higher-carbohydrate diet using corn starch and sucrose; HFHC-CS, high-fat higher-carbohydrate diet using corn starch and sucrose; HFHC-W, high-fat higher-carbohydrate diet using wheat; HFLC-W, high-fat, lower-carbohydrate, relatively higher-protein diet using wheat; HFHC-R, high-fat higher-carbohydrate diet using rice. The results are expressed as the means ± s.e.m. * *p* < 0.05, ‡ < 0.05, ** *p* < 0.01. Panels A and C: two-way repeated-measures ANOVA; panels B and D: one-way ANOVA, followed by Tukey’s multiple-comparisons test. The comparison symbols indicate the following comparisons: * HFHC-R versus HFHC-W or HFHC-CS; and ‡ LFHC-CS versus HFHC-CS.

Results from the OGTT showed that the rice-flour-based diet group (HFHC-R) exhibited significantly lower blood glucose levels at 90 and 120 min compared to the wheat-flour-based diet group (HFHC-W) and the corn starch/sucrose-based diet group (HFHC-CS) (Figure 2A). When comparing the area under the curve (AUC), we noted that the rice-flour-based diet group (HFHC-R) showed a significantly lower AUC compared to the wheat-flour-based diet group (HFHC-W) (*p* = 0.0483) and HFHC-CS (*p* = 0.0153) (Figure 2B). No significant difference in OGTT AUC was observed between the HFLC-W and HFHC-W groups (Figure 2B).

The results of the intraperitoneal insulin tolerance test (IPITT) showed that blood glucose levels 30 min after insulin administration were significantly higher in the high-fat diet group (HFHC-CS) compared to the low-fat diet group (LFHC-CS) (Figure 2C). When the rice-flour-based and wheat-flour-based diet groups were compared, the rice-flour-based diet was found to have significantly lower blood glucose levels at baseline and 30 min compared to the wheat-flour-based diet group (Figure 2C). No statistically significant difference was observed between the HFLC-W and HFHC-W groups (Figure 2C). No significant intergroup differences were observed in the AUC during the IPITT (Figure 2D).

The rice-flour-based diet was associated with improved glucose tolerance, as indicated by the significantly lower OGTT AUC. However, the results of the IPITT did not provide sufficient evidence to conclude that insulin sensitivity had improved. As there was variation in baseline blood glucose levels between groups in the IPITT, this may have affected the between-group comparisons based on absolute blood glucose levels.

### 3.3. Hepatic and Adipose Tissue Histology, Liver and Plasma Triglyceride Concentrations, and Mcp1 mRNA Expression in KK-A^y^ Mice Fed the Experimental Diets

**Figure 3:**
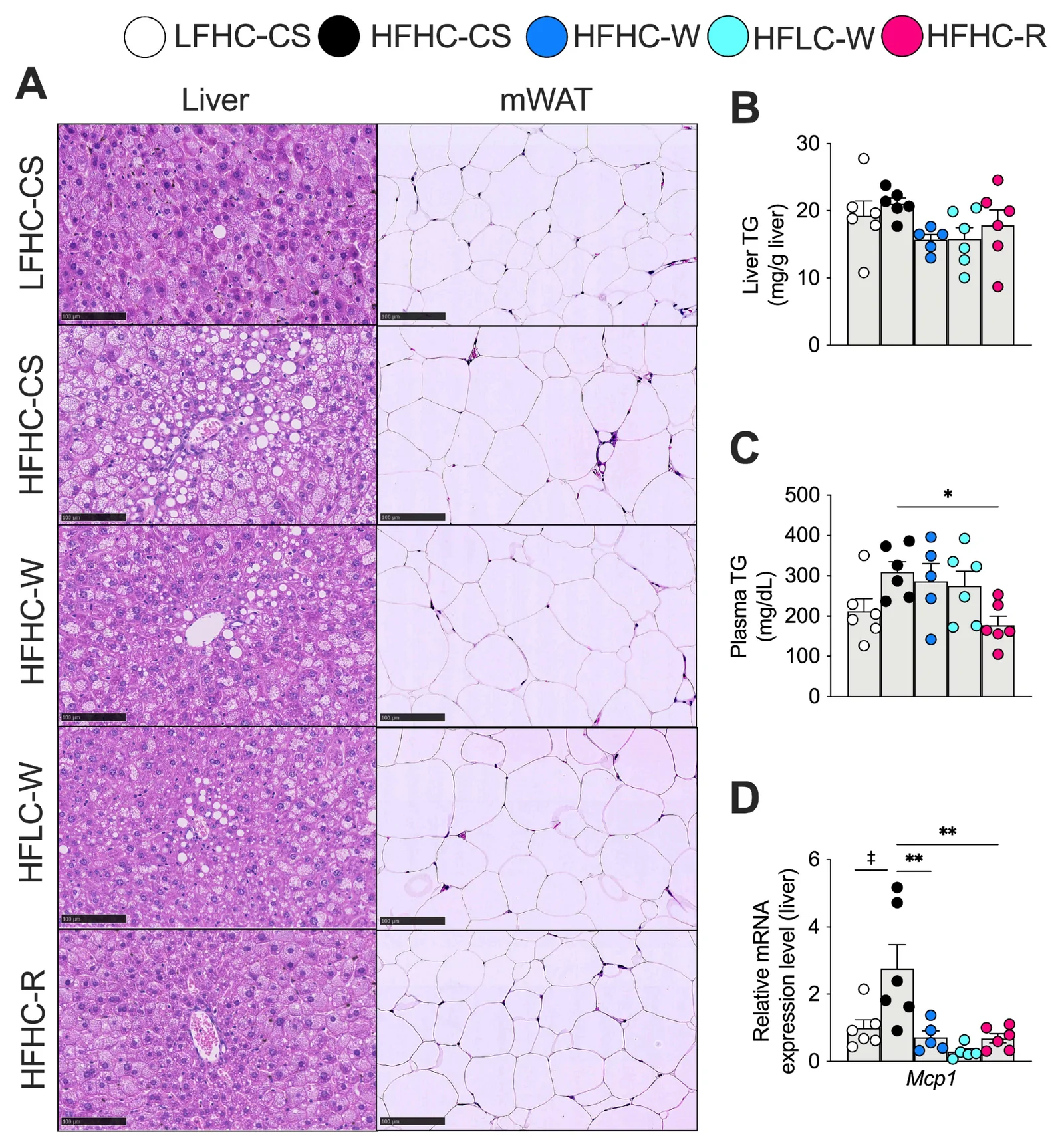
Hepatic and adipose tissue histology, liver and plasma triglyceride concentrations, and *Mcp1* mRNA expression in KK-*A^y^* mice fed the experimental diets. (**A**) Representative sections of liver tissue and mesenteric white adipose tissue stained with haematoxylin and eosin (H&E). Histological evaluation was performed using sections from three animals per group (*n* = 3 animals/group), and one representative image from one animal in each group is shown. Scale bar, 100 μm. (**B**) Liver TG (*n* = 5 for the HFHC-W group, and *n* = 6 for all other groups). (**C**) Plasma TG (*n* = 5 for the HFHC-W group, and *n* = 6 for all other groups). (**D**) Relative transcript levels of inflammation-related genes in the liver (*n* = 5 for the HFHC-W and HFLC-W group, and *n* = 6 for all other groups). One value in the HFLC-W group was excluded using the ROUT method (Q = 1%). LFHC-CS, low-fat higher-carbohydrate diet using corn starch and sucrose; HFHC-CS, high-fat higher-carbohydrate diet using corn starch and sucrose; HFHC-W, high-fat higher-carbohydrate diet using wheat; HFLC-W, high-fat, lower-carbohydrate, relatively higher-protein diet using wheat; HFHC-R, high-fat higher-carbohydrate diet using rice. The results are expressed as the means ± s.e.m. * *p* < 0.05, ‡ *p* < 0.05, ** *p* < 0.01. *p* values were calculated using analysis of variance, assessed by Tukey’s multiple comparisons test. The comparison symbols indicate the following comparisons: * HFHC-R versus HFHC-W or HFHC-CS; and ‡ LFHC-CS versus HFHC-CS.

Next, since lipid accumulation in organs is critical for obesity and its complications, we examined lipid accumulation in the liver and mWAT. Representative samples of these tissues were observed using haematoxylin and eosin (H&E) staining. H&E staining of the common section of the liver revealed qualitative differences in hepatic morphology and lipid accumulation among the groups. Within the high-fat diet groups, lipid accumulation in the liver qualitatively appeared lower in the rice-flour-based diet group (HFHC-R) compared to the wheat-flour-based diet group (HFHC-W) (Figure 3A). No obvious qualitative differences in hepatic lipid accumulation were observed when comparing the carbohydrate and protein quantity of the wheat-flour-based diet (HFHC-W vs. HFLC-W) (Figure 3A). In mWAT, adipocytes qualitatively appeared larger in the high-fat groups, whereas the low-carbohydrate, relatively higher-protein diet group (HFLC-W); the wheat-flour-based diet group (HFHC-W); and the rice-flour-based diet group (HFHC-R) showed a qualitative tendency toward smaller adipocytes compared with the corn-starch/sucrose-based diet group (HFHC-CS) (Figure 3A). Hepatic TG concentrations did not differ significantly among the groups, including between the HFHC-R and HFHC-W groups (Figure 3B). In contrast, serum TG concentrations were significantly lower in the HFHC-R group than in the HFHC-CS group (*p* = 0.0495), whereas no significant differences were observed between the HFHC-R and HFHC-W groups (Figure 3C). As liver lipid accumulation correlates with enhanced liver inflammation, gene expression analysis of *Mcp1* was performed, which showed that *Mcp1* mRNA expression was significantly higher in the HFHC-CS than in the LFHC-CS group (*p* = 0.0178) (Figure 3D). In contrast, compared to the corn-starch/sucrose-based diet group (HFHC-CS), *Mcp1* mRNA expression was significantly reduced in the wheat-flour-based diet group (HFHC-W) (*p* = 0.0081) and rice-flour-based diet group (HFHC-R) (*p* = 0.0046). The corn-starch/sucrose-based high-fat diet was associated with higher *Mcp1* mRNA expression (Figure 3D).

### 3.4. Expression of Genes Involved in Glucose Metabolism, Pyruvate Oxidation, and the TCA Cycle in Skeletal Muscle of KK-A^y^ Mice Fed the Experimental Diets

**Figure 4:**
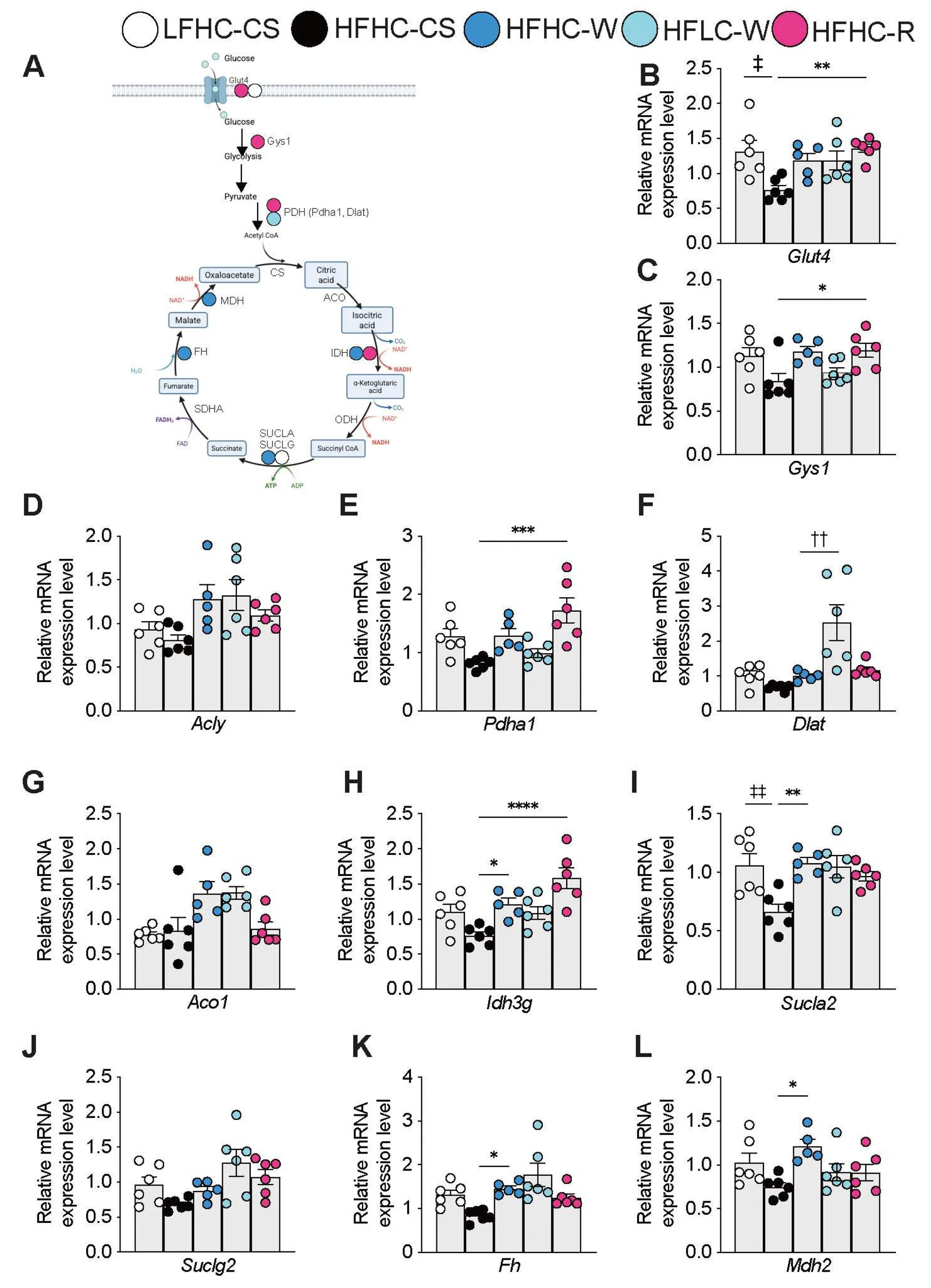
Expression of genes involved in glucose metabolism, pyruvate oxidation, and the TCA cycle in skeletal muscle of KK-*A^y^* mice fed the experimental diets. (**A**) TCA cycle and overview of the results (created with BioRender.com). (**B**) Relative transcript levels of *Glut4* genes in the skeletal muscle (*n* = 5 for the HFHC-W group, and n = 6 for all other groups). (**C**) Relative transcript levels of *Gys1* genes in the skeletal muscle (*n* = 5 for the HFHC-W group, and *n* = 6 for all other groups). (**D**–**L**) Relative transcript levels of genes encoding enzymes involved in pyruvate oxidation and the TCA cycle in the skeletal muscle (*n* = 5 for the HFHC-W group, and *n* = 6 for all other groups). LFHC-CS, low-fat higher-carbohydrate diet using corn starch and sucrose; HFHC-CS, high-fat higher-carbohydrate diet using corn starch and sucrose; HFHC-W, high-fat higher-carbohydrate diet using wheat; HFLC-W, high-fat, lower-carbohydrate, relatively higher-protein diet using wheat; HFHC-R, high-fat higher-carbohydrate diet using rice. The results are expressed as the means ± s.e.m. * *p* < 0.05, ‡ *p* < 0.01, ** *p* < 0.01, †† *p* < 0.01, ‡‡ *p* < 0.01, *** *p* < 0.001, and **** *p* < 0.0001. *p* values were calculated using analysis of variance, assessed by Tukey’s multiple comparisons test. The comparison symbols indicate the following comparisons: * HFHC-R versus HFHC-W or HFHC-CS; † HFHC-W versus HFLC-W; and ‡ LFHC-CS versus HFHC-CS.

Next, we focused on skeletal muscle, which is as important as adipose tissue for energy metabolism. We analysed the expression of genes involved in glucose transportation and glucose metabolism in skeletal muscle (Figure 4A). The expression of the glucose transporter *Glut4* showed a significant increase in the rice-flour-based group (HFHC-R) compared to the corn-starch/sucrose-based diet group (HFHC-CS) (*p* = 0.0070) and in the low-fat diet group (LFHC-CS) compared to the high-fat diet group (HFHC-CS) (*p* = 0.0140) (Figure 4B). Furthermore, the expression of *Gys1*, which encodes glycogen synthase, was significantly increased in muscle tissue of the rice-flour-based group (HFHC-R) compared to the corn-starch/sucrose-based diet group (HFHC-CS) (*p* = 0.0302) (Figure 4C). Although based on gene expression levels, *Glut4* and *Gys1* were consistently and significantly increased in the rice-flour-based diet group (HFHC-R) compared to the corn-starch/sucrose-based diet group (HFHC-CS) (Figure 4C). No significant differences were observed when comparing the rice-flour-based diet (HFHC-R) to the wheat-flour-based diet (HFHC-W) or in the comparison between the HFHC-W and HFLC-W groups, which differed in both carbohydrate and protein proportions (Figure 4C). The observed increase in *Glut4* mRNA expression may indicate altered regulation of GLUT4-mediated glucose metabolism; however, GLUT4 protein abundance, membrane translocation, and actual glucose uptake were not directly assessed.

Furthermore, examination of gene expression for genes encoding enzymes involved in pyruvate oxidation and the TCA cycle revealed that *Pdha1* (*p* = 0.0004) and *Idh3g* (*p* < 0.0001) were significantly increased in the rice-flour-based diet group (HFHC-R) compared to the corn-starch/sucrose-based diet group (HFHC-CS) (Figure 4D–L). In the wheat-flour-based diet group (HFHC-W), *Idh3g* (*p* = 0.0469), *Sucla2* (*p* = 0.0081), *Fh* (*p* = 0.0482), and *Mdh2* (*p* = 0.0128) mRNA expression were significantly increased compared to the corn/sucrose-based diet group (HFHC-CS) (Figure 4D–L). Although several genes differed relative to the HFHC-CS group, no significant differences were observed between the HFHC-R and HFHC-W groups (Figure 4D–L).

### 3.5. Expression of Genes Involved in Mitochondrial Energy Metabolism and the Electron Transport Chain in Skeletal Muscle of KK-A^y^ Mice Fed the Experimental Diets

**Figure 5:**
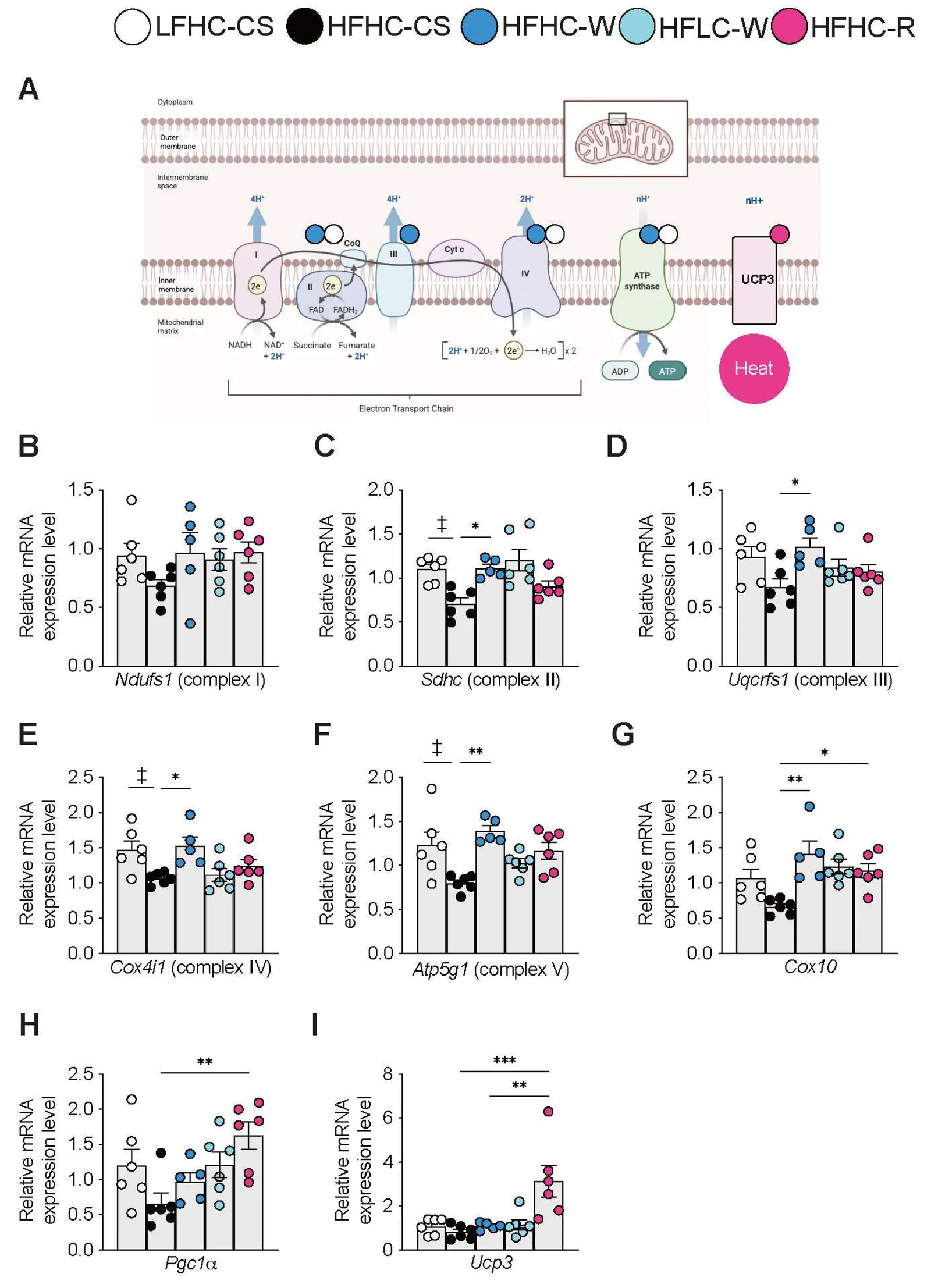
Expression of genes involved in mitochondrial energy metabolism and the electron transport chain in skeletal muscle of KK-*A^y^* mice fed the experimental diets. (**A**) Electron transport chain and overview of the results (created with BioRender.com). (**B**–**F**) Relative transcript levels of electron-transport-chain-related genes in the gastrocnemius muscle (*n* = 5 for the HFHC-W group, and *n* = 6 for all other groups). (**G**–**I**) Relative transcript levels of mitochondrion-related genes in the gastrocnemius muscle (*n* = 5 for the HFHC-W group, and *n* = 6 for all other groups). LFHC-CS, low-fat higher-carbohydrate diet using corn starch and sucrose; HFHC-CS, high-fat higher-carbohydrate diet using corn starch and sucrose; HFHC-W, high-fat higher-carbohydrate diet using wheat; HFLC-W, high-fat, lower-carbohydrate, relatively higher-protein diet using wheat; HFHC-R, high-fat higher-carbohydrate diet using rice. The results are expressed as the means ± s.e.m. * *p* < 0.05, ‡ < 0.05, ** *p* < 0.01, *** *p* < 0.001. *p* values were calculated using analysis of variance, assessed by Tukey’s multiple comparisons test. The comparison symbols indicate the following comparisons: * HFHC-R versus HFHC-W or HFHC-CS; and ‡ LFHC-CS versus HFHC-CS.

Gene expression analysis of key molecules in the electron transport system was then carried out to evaluate the expression of genes involved in mitochondrial energy metabolism and the electron transport chain (Figure 5A).

The mRNA expression of genes encoding components of complexes II (*p* = 0.0124), III (*p* = 0.0269), IV (*p* = 0.0245), and V (*p* = 0.0015) was significantly higher in the wheat-flour-based diet group compared with the corn-starch/sucrose-based diet group (Figure 5B–F). No significant differences in the expression of these electron-transport-chain-related genes were observed between the HFHC-R and HFHC-W groups.

COX10 is the gene encoding the Protoheme IX farnesyltransferase, a component of the mitochondrial Complex IV. The protein encoded by COX10 is an essential assembly factor for Cytochrome c oxidase (COX) synthesis and is involved in the first step of the mitochondrial Heme A biosynthesis pathway. Cox10 mRNA expression was significantly elevated in both the wheat-flour-based diet (HFHC-W) (*p* = 0.0016) and rice-flour-based diet (HFHC-R) (*p* = 0.0333) substitute groups compared with the corn/sucrose-based diet group (HFHC-CS) (Figure 5G).

Next, we focused on the transcription coactivator *Pgc1α*, which integrally regulates mitochondrial biogenesis. *Pgc1α* mRNA expression was significantly elevated only in the rice-flour-based diet group (HFHC-R) relative to the corn/sucrose-based diet group (HFHC-CS) (*p* = 0.0076) (Figure 5H). Furthermore, mRNA expression of the uncoupling protein *Ucp3* (Uncoupling Protein 3) was also significantly elevated only in the rice-flour-based diet group (HFHC-R) relative to the wheat-flour-based diet group (HFHC-W) (*p* = 0.0059) and corn/sucrose-based diet group (HFHC-CS) (*p* = 0.0010) (Figure 5I).

Skeletal muscle is the organ with the highest glucose uptake and plays a crucial role in metabolism [23]. The increased *Ucp3* mRNA expression may be associated with the observed lower body weight; however, UCP3 protein abundance, mitochondrial respiration, and whole-body energy expenditure were not assessed, and a causal role of UCP3 remains to be established.

## 4. Discussion

In this study using KK-*A^y^* mice—a model of obesity and type 2 diabetes without genetic abnormalities in the appetite-suppressing hormone leptin [19,20]—body weight was 8.5% lower in the rice-flour-based diet group than in the wheat-flour-based diet group after seven weeks. Specifically, the rice-flour-based diet was associated with significantly improved glucose tolerance, as evidenced by a reduction in the glucose AUC during the OGTT. In contrast, the glucose AUC during the IPITT did not differ significantly between the groups; therefore, the present findings do not provide sufficient evidence of improved insulin sensitivity. Baseline differences in glucose levels may have influenced the IPITT results. Furthermore, GLUT4 protein expression, GLUT4 translocation, and glucose uptake in insulin-responsive tissues were not directly assessed. Therefore, the mechanisms underlying the observed improvement in glucose tolerance remain unclear, and further studies are required to determine whether altered peripheral glucose uptake contributes to this effect. In H&E-stained sections, although lipid accumulation in the livers of the rice-flour-based diet group appeared to be qualitatively lower than that in the wheat-flour-based diet group, no significant differences were observed in hepatic TG concentrations between the two groups. This seemingly contradictory result may be attributable to the qualitative nature of the histological assessment, localized heterogeneity in lipid distribution within the liver, or differences between the endpoints assessed histologically and those assessed by biochemical triglyceride quantification. Therefore, the results of this study alone do not provide sufficient evidence to conclude that the rice-flour-based diet reduced lipid accumulation in the liver.

In contrast, when comparing diets containing wheat with different carbohydrate and protein levels (energy ratios of 35% versus 45% of carbohydrates and 20% versus 10% of protein, respectively), no statistically significant differences in body weight were observed between the HFLC-W and HFHC-W groups under the conditions of the present study. This may reflect limited statistical power rather than biological equivalence.

In a previous study in which a rice-flour-based diet and a wheat-flour-based diet were administered to rats, the components other than the carbohydrate source were standardized in the same way as in the present study, and the control group’s corn starch, sucrose, and maltodextrin were replaced with rice-flour or wheat-flour. As a result, compared with the corn starch, sucrose, and maltodextrin group, a significant rise in blood glucose levels was observed only in the wheat-flour-based diet group; consistent with the results of this study, the rice-flour-diet group showed a more favourable blood glucose response than the wheat-flour-based diet group [24].

Several previous studies indicating that dietary grain may affect blood glucose levels have compared whole grains with refined grains [25,26] or the amount of resistant starch [27]. A comparison of rice flours—which differ in resistant starch content, even within the same grain type, due to varietal differences—showed that rice with a high resistant starch content resulted in a significantly greater reduction in fat weight than rice with a low resistant starch content [27]. In this study, the same amount of dietary fibre—which directly influences the gut microbiota—was added to all groups. Furthermore, the wheat flour contained a higher amount of resistant starch at the ingredient level (Table A2). Consistent with this difference, the resistant starch content values of the final HFHC-R and HFHC-W diets were 2.65 and 7.50 mg/g diet, respectively. The estimated daily resistant starch intake was 16.21 mg/mouse/day in the HFHC-R group and 46.52 mg/mouse/day in the HFHC-W group. Consequently, mice in the wheat-flour-based diet group consumed more resistant starch than those in the rice-flour-based diet group. Whilst this difference should be regarded as a potential dietary confounder, a higher intake of resistant starch is generally expected to lead to reduced weight gain and improved glucose metabolism. Consequently, it is unlikely that the high intake of resistant starch in the wheat-flour-based diet group explains the observed weight gain or impaired glucose metabolism; rather, it may have mitigated these differences between the groups. Thus, resistant starch and dietary fibre differences alone are unlikely to provide a sufficient explanation of the observed group differences. In future studies, it will be necessary to examine differences in the structure of the starch in rice flour and wheat flour (the ratio of amylose to amylopectin) [28], as well as differences in the types and content of phenolic compounds [29], flavonoids [30], and lipid components [24], amongst other factors.

In this study, organ-specific gene expression analysis revealed that replacing a wheat-flour-based diet with a rice-flour-based diet was associated with increased *Ucp3* mRNA expression in skeletal muscle. UCPs present in mitochondria are specific proteins that uncouple oxidative phosphorylation, converting its energy into heat [31]. Previous studies showed that *Ucp3* transgenic mice exhibited a lean phenotype despite overeating [32,33] and had reduced ATP production in skeletal muscle mitochondria [33]. This result corresponds to a roughly 42% decrease in mitochondrial oxidative phosphorylation efficiency, indicating that overexpression of *Ucp3* in skeletal muscle significantly promotes energy expenditure [33]. Our findings are consistent with the increased *Ucp3* mRNA expression and the lower body weight observed in the rice-flour-based diet group. The increased *Ucp3* mRNA expression may be associated with the observed phenotype; however, whether UCP3 contributes causally to the reduction in body weight remains to be determined. Also, because the wheat-flour-based and rice-flour-based diet groups were not completely matched in their overall nutrient and bioactive profiles—differing in protein, lipid, resistant starch, and phenolic compounds—it remains unclear whether this upregulation of *Ucp3* mRNA expression is attributable to specific starch characteristics of rice or to other confounding dietary constituents. Therefore, these molecular changes should be interpreted as a response to the whole-diet substitution rather than an isolated effect of rice starch itself.

Furthermore, although several genes involved in pyruvate oxidation and the TCA cycle showed altered expression relative to the HFHC-CS group, no significant differences were observed between the rice-flour-based and wheat-flour-based diet groups. The observed changes may be consistent with altered metabolic activity; however, whether these changes reflect an acceleration of metabolic turnover remains speculative and would require direct assessment using metabolic-flux, energy-expenditure, oxygen consumption and physical activity measurements.

Furthermore, a systematic review suggests that *Ucp3* mRNA expression changes may be involved in weight loss following bariatric surgery, one of the most effective surgical therapies for patients with severe obesity [34]. Moreover, skeletal muscle UCP3 was reduced in patients with diabetes [35]. The potential relevance of *Ucp3* to human metabolism is further suggested by its identification as a candidate gene for hereditary obesity susceptibility in the Human Obesity Gene Map [36,37,38].

Potential factors promoting an increase in *Ucp3* expression include non-esterified fatty acids [39,40], superoxide anion [39,40], thyroid hormone [41], 9-cis retinoic acid [42,43], leptin [41], and irisin [44]. However, specific inducers of *Ucp3* expression have yet to be identified, and our gene expression data alone do not establish a definitive metabolic pathway. While the results suggest a potential shift toward increased energy expenditure, direct assessment of UCP3 protein abundance, mitochondrial respiration and whole-body energy expenditure would be required to substantiate this proposed mechanism.

The first limitation of this study is that the composition of the wheat flour and rice flour (including the content of carbohydrates, protein, lipids and other bioactive substances) was not consistent across the groups. The present study does not provide sufficient evidence to determine whether the observed improvements in body weight and glucose metabolism are attributable to rice flour as a whole or to a specific component of rice flour. Consequently, it should be noted that the results of this study reflect differences between the wheat-flour-based diet and rice-flour-based diet and do not identify specific active substances such as wheat starch or rice starch.

The second limitation concerns the effects of heating and freeze-drying on starch properties. Different heating methods were used for the rice and wheat materials, and these procedures may have differentially affected starch gelatinization, crystallinity, amylose–lipid interactions, retrogradation, resistant starch content, and digestibility. As these starch properties were not characterized after processing, we cannot exclude the possibility that processing-induced changes contributed to the observed metabolic effects. Further studies using compositionally matched diets and standardized processing conditions, together with post-processing characterization of starch properties, are required to identify the components and mechanisms responsible for these effects.

The third limitation of this study is that only female mice were used, and caution should be exercised when generalizing. Concerning the impact of sex differences on glucose and lipid metabolism, oestrogen, a female hormone, has been reported to have a protective effect against insulin resistance, providing resistance against obesity and diabetes [45]. The KK-*A^y^* mouse is a specific model in which both males and females develop obesity and diabetes; however, females are more prone to obesity, whilst males are more prone to insulin resistance [46]. Previous studies using other mouse strains have also reported sex- and hormone-associated variation in *Ucp3* mRNA expression [47]. However, because oestrogen concentrations and oestrous-cycle stage were not assessed in the present study, potential effects of sex hormone status and oestrous-cycle variation on *Ucp3* mRNA expression and metabolic outcomes cannot be excluded. These factors represent potential sources of biological variation and should be investigated in future studies.

The fourth limitation of this study concerns the setting of the proportion of carbohydrates in the diet. Regarding the carbohydrate ratio, meta-analyses often use a cut-off of >45% of calories for high carbohydrates and ≦45% for low carbohydrates [48,49]. The present study compared 45% and 35%, constituting a cut-off similar to that used in previous studies for high carbohydrates and low carbohydrates. Because carbohydrate and protein proportions differed simultaneously and the sample size was small, this study cannot determine whether the non-significant result reflects an insufficient macronutrient difference or limited statistical power.

The fifth limitation of this study is the sample size. We used a relatively small number of animals (*n* = 5–6 mice per group) to investigate a large number of physiological, biochemical, histological and molecular endpoints. As no prior justification of the sample size or formal power calculations were performed, this study may have had limitations in terms of statistical power. Consequently, specifically in cases where no significant differences were observed—such as in the comparison of body weight between the HFLC-W and HFHC-W groups—the absence of a significant difference may be attributable to insufficient statistical power rather than the true absence of an effect.

Furthermore, in the gene expression analysis, multiple statistical comparisons were performed without adjusting the significance level. As this study was designed as an exploratory and hypothesis-generating investigation rather than a confirmatory trial, strict multiple-comparison corrections, such as the Benjamini–Hochberg method, were not applied to the RT-PCR dataset. This approach was chosen to minimize the risk of false negatives and to ensure that potentially important biological signals were not overlooked. However, this statistical strategy inevitably increases the risk of acquiring false-positive results. Consequently, isolated significant differences in mRNA expression must be interpreted with due caution, and these molecular changes should be regarded as potential exploratory associations rather than definitive causal mechanisms.

## 5. Conclusions

In this mouse model, the rice-flour-based diet was associated with an 8.5% lower body weight and improved glucose tolerance compared with the wheat-flour-based diet. Because the composition of the diet’s major nutrients and bioactive components was not consistent across the groups, these findings should be interpreted as reflecting differences between the wheat-flour-based diet and rice-flour-based diet as a whole rather than the type of starch itself. Moreover, because this study was conducted exclusively on female KK-*A^y^* mice, the findings cannot be directly generalized to males, other animal models, or humans and should not be interpreted as evidence of clinical efficacy. Further studies should include detailed compositional comparison of the wheat flour and rice flour used and investigate the effects of individual components to identify the constituents responsible for the observed metabolic outcomes. Also, direct evaluation of energy expenditure, oxygen consumption or physical activity, and mitochondrial function, alongside verification through trials using other models (including both males and females) and, ultimately, human subjects, will be necessary for future studies.

## Data Availability

The original contributions presented in this study are included in the article. Further inquiries can be directed to the corresponding authors.

## Abbreviations

The following abbreviations are used in this manuscript:

*Ucp3*	Uncoupling Protein 3
GI	Glycaemic index
LFHC-CS	The low-fat and higher-carbohydrate (corn starch and sucrose) diet group
HFHC-CS	The high-fat and higher-carbohydrate (corn starch and sucrose) diet group
HFHC-W	The high-fat and higher-carbohydrate (wheat) group
HFLC-W	The high-fat and lower-carbohydrate, relatively higher-protein (wheat) diet group
HFHC-R	The high-fat and higher-carbohydrate (rice (Koshihikari)) diet group
OGTT	Oral glucose tolerance test
IPITT	Intraperitoneal insulin tolerance test
AGPC	Acid guanidinium thiocyanate–phenol–chloroform extraction
AUC	Area under the curve
H&E	Haematoxylin and eosin
COX	Cytochrome c oxidase

## Appendix A

**Table A1:** Diet composition.

LFHC-CS					
Nutrient	Ingredient	Amount (g)	Energy (kcal)	Energy (kcal%)	Macronutrient Energy (kcal%)
Protein	Casein, Lactic	100	400	9.7%	
Protein	Cystine	3	12	0.3%	10.0%
Carbohydrate	Sucrose	176.8	707.2	17.2%	
Carbohydrate	αstarch, Corn	100	400	9.7%	
Carbohydrate	Starch, Corn	547.2	2188.8	53.1%	80.0%
Fat	Lard	20.8	187	4.5%	
Fat	Soybean Oil	25	225	5.5%	10.0%
Mineral	AIN-93G-MX	35			
Vitamin	Vitamin Mix	10			
Total		1017.8	4120		
**HFHC-CS**					
**Nutrient**	**Ingredient**	**Amount (g)**	**Energy (kcal)**	**Energy (kcal%)**	**Macronutrient Energy (kcal%)**
Protein	Casein, Lactic	100	400	9.9%	
Protein	Cystine	3	12	0.3%	10.2%
Carbohydrate	Sucrose	176.8	707.2	17.4%	
Carbohydrate	αstarch, Corn	100	400	9.9%	
Carbohydrate	Starch, Corn	178.825	715.3	17.6%	44.9%
Fat	Lard	177.5	1597.5	39.4%	
Fat	Soybean Oil	25	225	5.5%	44.9%
Mineral	AIN-93G-MX	35			
Vitamin	Vitamin Mix	10			
Total		806.125	4057		
**HFHC-W**					
**Nutrient**	**Ingredient**	**Amount (g)**	**Energy (kcal)**	**Energy (kcal%)**	**Macronutrient Energy (kcal%)**
Protein	Casein, Lactic	100	400	9.9%	
Protein	Cystine	3	12	0.3%	10.2%
Carbohydrate	Wheat	176.8	707.2	17.4%	
Carbohydrate	αstarch, Corn	100	400	9.9%	
Carbohydrate	Wheat	178.825	715.3	17.6%	44.9%
Fat	Lard	177.5	1597.5	39.4%	
Fat	Soybean Oil	25	225	5.5%	44.9%
Mineral	AIN-93G-MX	35			
Vitamin	Vitamin Mix	10			
Total		806.125	4057		
**HFLC-W**					
**Nutrient**	**Ingredient**	**Amount (g)**	**Energy (kcal)**	**Energy (kcal%)**	**Macronutrient Energy (kcal%)**
Protein	Casein, Lactic	200	800	19.8%	
Protein	Cystine	3	12	0.3%	20.1%
Carbohydrate	Wheat	176.8	707.2	17.5%	
Carbohydrate	αstarch, Corn	100	400	9.9%	
Carbohydrate	Wheat	72.8	291.2	7.2%	34.7%
Fat	Lard	177.5	1597.5	39.6%	
Fat	Soybean Oil	25	225	5.6%	45.2%
Mineral	AIN-93G-MX	35			
Vitamin	Vitamin Mix	10			
Total		800.1	4032.9		
**HFHC-R**					
**Nutrient**	**Ingredient**	**Amount (g)**	**Energy (kcal)**	**Energy (kcal%)**	**Macronutrient Energy (kcal%)**
Protein	Casein, Lactic	100	400	9.9%	
Protein	Cystine	3	12	0.3%	10.2%
Carbohydrate	Rice	176.8	707.2	17.4%	
Carbohydrate	αstarch, Corn	100	400	9.9%	
Carbohydrate	Rice	178.825	715.3	17.6%	44.9%
Fat	Lard	177.5	1597.5	39.4%	
Fat	Soybean Oil	25	225	5.5%	44.9%
Mineral	AIN-93G-MX	35			
Vitamin	Vitamin Mix	10			
Total		806.125	4057		

**Table A2:** Grain food compositions.

(g/100 g)	Rice Flour	Wheat Flour
Protein	6.0	8.3
Isoleucine (mg)	240	320
Leucine (mg)	490	610
Lysine (mg)	200	190
Methionine (mg)	140	150
Cystine (mg)	140	240
Phenylalanine (mg)	320	450
Tyrosine (mg)	280	270
Threonine (mg)	220	260
Histidine (mg)	150	200
Tryptophan (mg)	85	110
Valine (mg)	360	380
Fat	0.7	1.5
Saturated Fatty Acids	0.25	0.34
Monounsaturated Fatty Acids	0.12	0.13
Polyunsaturated Fatty Acids	0.2	0.75
n-3 Polyunsaturated Fatty Acids	0.01	0.04
n-6 Polyunsaturated Fatty Acids	0.2	0.72
Carbohydrate	81.9	75.8
Dietary Fiber	0.6	2.5
Water-soluble	trace	1.2
Water-insoluble	0.6	1.3
Starch	74.2	72.7
Resistant starch *	0.6	1.7

Based on Standard Tables of Food Composition in Japan (Eighth Revised Edition) (Updated and Enlarged Version 2023). * Megazyme resistant starch assay kit (Megazyme International Ireland Ltd., Wicklow, Ireland).

**Table A3:** Primer sequences.

Genes	Forward Primers (5′→3′)	Reverse Primers (5′→3′)
***18S***	TTCTGGCCAACGGTCTAGACAAC	CCAGTGGTCTTGGTGTGCTGA
***β-actin***	CATCCGTAAAGACCTCTATGCCAAC	ATGGAGCCACCGATCCACA
***Mcp1***	CCACTCACCTGCTGCTACTCAT	TGGTGATCCTCTTGTAGCTCTCC
***Glut4***	CTGTAACTTCATTGTCGGCATGG	AGGCAGCTGAGATCTGGTCAAAC
***Acly***	GTGGCCCCAACTATCAAGAG	AATGGCCGTCATGTGAGTTT
***Pdha1***	AAGATGCTTGCCGCTGTATC	AGCCGATGAAGGTCACATTT
***Dlat***	GCAGCAGAGAAAGGGATTGA	AGCAGCCTTAGAAGGCACAA
***Aco1***	TTCGGGCCAGGAGTGGCTCA	CTTCCTCGCGGCCTGTCTGC
***Idh3g***	AACTCTCCTCTGCCGTCCTT	TATGCCGCCCACCATACTTA
***Sucla2***	CGCAAATATCCCAGGAGAGA	CACCACCTTGTGCACTTCC
***Suclg2***	TGGTGTAAAGGAAGCCCAAG	AGTTGACGATCCCACCAAAG
***Fh***	TGCATATTGCTGCTGCAGTGGAAG	ACCATCGCATACTGGACTTGCTGA
***Mdh2***	GGCCAAGGCTGGAGCAGGTTC	TCATGGCGTCCACGAGGGAGA
***Ndufs1***	GGAACTACTCGGTGGGCTC	GCCAGTTGTGCGAACATATC
***Sdhc***	AGTTTGTGCTTGTCTTCCCG	CACTCCAGACAGCCAGACCT
***Uqcrfs1***	ATGTGAAGCGACCCTTCCT	GGAAAAACGGACAGAAGCAG
***Cox4i1***	GAGCCTGATTGGCAAGAGAG	GATCAGCGTAAGTGGGGAAA
***Atg5g1***	CACTGCTCATTTCTCCAGCTC	CAGGAAGGCTGCTTAGATGG
***Cox10***	TTCCTGCTTACATCCCTTGG	TTCATGTTTGAGTCGAACGG
***Pgc1a***	AAGGGCCAAACAGAGAGAGA	GCGTTGTGTCAGGTCTGATT
***Ucp3***	CTCTGCACTGTATGCTGAAGATG	CACGTTCCAAGCTCCCAGA
